# Lung epithelial‐specific TRIP‐1 overexpression maintains epithelial integrity during hyperoxia exposure

**DOI:** 10.14814/phy2.13585

**Published:** 2018-02-27

**Authors:** Michael F. Nyp, Sherry M. Mabry, Angels Navarro, Heather Menden, Ricardo E. Perez, Venkatesh Sampath, Ikechukwu I. Ekekezie

**Affiliations:** ^1^ Division of Neonatology Department of Pediatrics Children's Mercy Kansas City Kansas City Missouri; ^2^ Department of Pediatrics University of Missouri Kansas City Kansas City Missouri; ^3^ Department of Anatomy and Cell Biology Rush University Chicago Illinois

**Keywords:** Airway and lung biology, hyperoxic acute lung injury, TGFβ, TRIP‐1

## Abstract

The onset and degree of injury occurring in animals that develop hyperoxic acute lung injury (HALI) is dependent on age at exposure, suggesting that developmentally regulated pathways/factors must underlie initiation of the epithelial injury and subsequent repair. Type II TGFβ receptor interacting protein‐1 (TRIP‐1) is a negative regulator of TGFβ signaling, which we have previously shown is a developmentally regulated protein with modulatory effects on epithelial‐fibroblastic signaling. The aim of this study was to assess if type II alveolar epithelial cells overexpressing TRIP‐1 are protected against hyperoxia‐induced epithelial injury, and in turn HALI. Rat lung epithelial cells (RLE) overexpressing TRIP‐1 or LacZ were exposed to 85% oxygen for 4 days. A surfactant protein C (SPC)‐driven TRIP‐1 overexpression mouse (TRIP‐1^AECTg+^) was generated and exposed to hyperoxia (>95% for 4 days) at 4 weeks of age to assess the effects TRIP‐1 overexpression has on HALI. RLE overexpressing TRIP‐1 resisted hyperoxia‐induced apoptosis. Mice overexpressing TRIP‐1 in their lung type II alveolar epithelial cells (TRIP‐1^AECTg+^) showed normal lung development, increased phospho‐AKT level and E‐cadherin, along with resistance to HALI, as evidence by less TGFβ activation, apoptosis, alveolar macrophage influx, KC expression. Taken together, these findings point to existence of a TRIP‐1 mediated molecular pathway affording protection against epithelial/acute lung injury.

## Introduction

Hyperoxic acute lung injury (HALI) is a consequence of prolonged exposure to high concentrations of oxygen and carries a high degree of morbidity and mortality. Histological features of HALI include alveolar capillary leak, activation of inflammatory cells, and the development of hyaline membranes, with epithelial cell injury playing a key role (Zaher et al. 2007; Kallet and Matthay 2013). Central to epithelial cell injury is the effect supraphysiological oxygen exposure has on multiple intracellular signaling cascades, inducing apoptosis in type II alveolar epithelial cells (AEC). While type II AEC are essential for maintaining homeostatic pulmonary function under normal physiological conditions, hyperoxia results in loss of epithelial integrity through increased apoptosis signaling and possibly enhanced epithelial transdifferentiation (Kalluri and Neilson 2003; Leight et al. 2012). Loss of epithelial cell integrity with resulting injury contributes to cytokine release and enhanced immune cell recruitment observed in HALI (Barazzone et al. 1998).

Onset and degree of HALI is dependent on animal age at exposure, the duration, and concentration of oxygen used. While most neonatal mouse pups survive high concentration of oxygen for up to 2 weeks, adult mice exposed to high concentrations of oxygen do not survive beyond 4–5 days of similar oxygen exposure (Frank et al. 1978). Additionally, the injury pattern occurring in neonatal mouse pups shows more alveolar simplification in contrast to adult mice showing hallmark features of HALI. These observations suggest developmentally regulated factors play a role in initiation of hyperoxia‐induced epithelial injury and subsequent repair.

Type II TGFβ receptor interacting protein‐1 (TRIP‐1) is a developmentally expressed endogenous protein and functional component of eukaryotic translation initiation factor‐3 (eIF3)(Asano et al. 1997; Chen et al. 2003). TRIP‐1 associates with, and is phosphorylated by, the TGFβ type II receptor kinase, and is capable of modulating TGFβ1 responses (Choy and Derynck 1998). However, TRIP‐1 has also been shown to interact with other factors, suggesting unrecognized roles of TRIP‐1 (Metz‐Estrella et al. 2012; Yuan et al. 2014; Ramachandran et al. 2016). In fibroblasts, our lab has shown TRIP‐1 is highly expressed in fetal fibroblasts and is negative regulator of fibroblast contractility and TGFβ signaling (Navarro et al. 2009; Nyp et al. 2014). Additionally, we have reported that TRIP‐1 is a key regulator of EMT signaling in epithelial cells treated with exogenous TGFβ in vitro (Perez et al. 2011). However, the role TRIP‐1 might play in hyperoxia‐induced epithelial injury, of which TGFβ signaling has been implicated as a critical player, has yet to be reported. On the basis of our studies, we hypothesized that epithelial‐specific TRIP‐1 overexpression will protect epithelial cells from hyperoxia‐mediated epithelial cell injury. To test this hypothesis, we evaluated the role TRIP‐1 overexpression plays in hyperoxia injury in rat lung epithelial (RLE) cells and generated a new SPC‐driven TRIP‐1 overexpression transgenic mouse. In this study, we show that TRIP‐1 overexpression protects RLE and isolated mouse type II AEC, from hyperoxia‐induced apoptosis, and maintains epithelial cell integrity, without significantly altering mesenchymal markers, during acute exposure. Transgenic mice overexpressing TRIP‐1 in type II AEC reduced histological findings of HALI.

## Material and Methods

### Transgenic construct

An expression plasmid containing a 3.7 Kb portion of the 5′ human SPC gene promoter cloned into pUC18 that has been used successfully to develop transgenic mouse models with expression directed to type II epithelial cells was obtained from Dr. Jeffrey Whitsett (Cincinnati Children's Hospital). The human TRIP‐1 DNA construct previously described in the production of TRIP‐1 overexpression epithelial and fibroblast cell lines was inserted into the SP‐C plasmid (Perez et al. 2011; Nyp et al. 2014). The TRIP‐1 overexpressing transgenic mouse with specific targeting was made at the University of Kansas Medical Center Transgenic Facility in an F1 (FVB females with C57Bl/6 males) background. Founding TRIP‐1 transgenic mice were confirmed by tailsnip genotyping and Southern blotting, and were bred with C57Bl/6 mice for at least 5 generations to return to a C57Bl/6 background, genotyping every generation using PCR analysis.

### Animal care

All animal studies were approved by the Institutional Animal Care and Use Committee at the University of Missouri‐Kansas City. TRIP‐1 transgenic, nontransgenic siblings and WT C57Bl/6 mice were housed in individually ventilated cages with food and water available ad libitum in a 12:12 dark/light environment.

### Animal exposure, sacrifice, and sample collection

Overexpressing or nonoverexpressing siblings were randomized at 4 weeks of age to either >95% O_2_ exposure or room air. For the hyperoxia exposure, mice were placed in cages in a Plexiglass chamber (36” x 24” x 16”) for 4 days and for a subset of mice up to 7 days. The enclosure was flushed with oxygen and the concentration was continuously monitored and controlled with a Proox oxygen controller (BioSpherix, Lacona, NY). Oxygen was infused at a rate of 10 L/min, as needed, to maintain >95% O_2_ throughout the study period. Mouse cages were briefly removed daily to assess mice and for cage maintenance. Control mice were housed similarly, but were not exposed to hyperoxia.

After 4 days, mice were killed and lungs were harvested for histology or protein and RNA analysis, or were lavaged with fluid collected for analysis. A separate group of mice were used for type II AEC isolation after 3 days of exposure.

### Histology and immunohistochemistry

The trachea was cannulated with a 19‐gauge blunt needle which was tied into place and attached to a stopcock. Lungs were inflated to 24 cm H_2_O pressure with Carson's Millonig Formalin (Fisher Scientific, Kalamazoo, MI) and placed in the fixative overnight, then processed and embedded in paraffin. Sections (5 *μ*m) were cut from the left lung and each lobe of the right lung and stained for hematoxylin and eosin according to established protocols, or immunohistochemically stained with the following: goat polyclonal antibody against CD68 (Santa Cruz Biotechnology, Dallas, TX) (1:50 dilution); mouse monoclonal V5 epitope tag antibody (ThermoFisher Scientific) (1:200 dilution); or a rabbit polyclonal antibody against TGFβ1 and phosphorylated Smad2. Peroxidase polymer detection kits were used for the CD68 and TGFβ1 stains (antigoat IgG and antirabbit IgG, respectively) and a mouse‐on‐mouse kit was used for the V5 stain (Vector Laboratories, Burlingame, CA). Antigenic sites were visualized with 3, 3′‐daminobenzidine and all sections were steamed for 20 min in citrate buffer, pH 6 (DAKO, Carpenteria, CA), prior to antibody application, to unlock antigenic sites.

Radial alveolar counts (RAC) were done at 100× with an Olympus BX60 microscope using previously described methods (Emery and Mithal 1960). Seven sections were examined for each mouse and an average of 22 fields/mouse was counted. A minimum of 15 high power (600X) fields (HPF) per mouse were examined to quantify CD68 staining, averaging the number of CD68‐positive cells/HPF. TGFβ1 staining was quantified at 200× magnification for TGFβ‐positive cells/field for a minimum of 15 fields per mouse. For pSMAD2 staining, nuclear pSMAD2 was quantified at 400× magnification on parenchyma excluding bronchial epithelium for nuclear staining and % nuclear expressing cells was tabulated on a minimum of 3–4 fields per mouse.

### Lung harvest for protein and RNA analysis

Phosphate‐buffered saline (5 mL) was perfused through the pulmonary vasculature via the right ventricle of the heart to remove blood. The left lung was minced and placed in 0.5 mL RNAlater for RNA analysis. The right lung was flash frozen in liquid nitrogen, and then stored at −80°C for western blot analysis.

### Western blot

Total lung or cell lysates were prepared using previously described methods (Navarro et al. 2009; Perez et al. 2011; Nyp et al. 2014). Western blots were developed using ECL (chemiluminescence) with the following antibodies: polyclonal rabbit anti‐TRIP‐1 (1:1000, Abcam); mouse monoclonal antismooth muscle actin (1:1000) and tubulin (1:10,000) (Sigma‐Aldrich); rabbit monoclonal antiphosphorylated Akt (1:1000) and rabbit polyclonal anti‐Akt (1:1000) (Cell Signaling); mouse monoclonal anti‐E‐cadherin (1:1000, BD Biosciences); and goat polyclonal antiactin (1:2000, Santa Cruz Biotechnologies).

### RNA isolation and real‐time PCR

Total RNA isolation was performed with TRIzol reagent according to the manufacturer's protocol. For mouse mRNA, the following primers were used:

mKC (IL‐8) F CAATGAGCTGCGCTGTCAGTG

mKC (IL‐8) R CTTGGGGACACCTTTTAGCATC

For rat mRNA, the following primer sequences were used:

GRO/CINC‐1 F: CATTAATATTTAACGATGTGGATGCGTTTCA,

GRO/CINC‐1 R: GCCTACCATCTTTAAACTGCACAA

Relative gene expression was determined by normalizing to GAPDH by the ^ΔΔ^Ct method. For mouse lung tissue RNA analysis, KiCqStart system from Sigma‐Aldrich was used to generate primers. For rat cell RNA analysis, reactions were run on a real‐time PCR system (iCycler MyiQ System; Bio‐Rad Laboratories, Hercules, CA), using iQ SYBR Green (Bio‐Rad) for gene detection. First‐strand cDNA synthesis was carried out with SuperScript III (Invitrogen, Carlsbad, CA).

### Bronchoalveolar lavage fluid collection

The mouse lungs were lavaged 10× with 200 *μ*L of phosphate‐buffered saline (PBS) using standard techniques. A 10 *μ*L aliquot was loaded on a hemacytometer for cell number counting and a 200 *μ*L aliquot was collected on a cytospin at room temperature for Diffquick staining.

### Isolation of alveolar type II epithelial cells from mouse lungs

Isolation of type II epithelial cells from mouse lungs was performed using the method developed by Corti et al. (1996) and Rice et al. (2002) with some modifications. Lungs were perfused with 10 mL PBS via the right ventricle, the trachea was cannulated, and dispase (3 mL; Fisher, Cincinnati, OH) was rapidly instilled followed by 0.5 mL warm 1% low melting point agarose in water. After hardening of the agarose (with ice, about 2 min), lungs were dissected and incubated in a tube containing 2 mL of dispase for 45 min, after which lung tissue was teased apart in a 60 mm dish containing 7 mL DMEM‐25 mmol/L HEPES and 100 U/mL DNAse I and incubated for an extra 5 min with swirling. After filtering through 100 *μ*m, 40 *μ*m, and 20 *μ*m nylon strainers, solution was spun at 130*g* for 8 min at 4°C, resuspended in 10 mL of DMEM/HEPES containing 10% FBS and 1% Pen‐Strep and allowed to attach to rat antimouse CD45/CD32‐coated dishes for 2 h at 37°C. After that time, the supernatant containing the epithelial cells was carefully removed, and was spun again at 130*g* for 8 min at 4°. Cells were resuspended in 1 mL DMEM/HEPES media, counted, and used to prepare cytospins for staining, or were collected for cell lysate preparation.

### Cell lines

RLE‐6TN cells were purchased from ATCC and grown in recommended conditions. For hyperoxia exposure, cells were plated at 200,000 cells/60 mm density and exposed after 24 h to a mixture of 85% O_2_/5% CO_2_, 10% N_2_ in a humidified chamber (Billups‐Rothenberg, Del Mar, CA), with the chamber flushed at a flow rate of 10 L/min for 15 min before incubation at 37°C. Cells were transfected and clones generated using previously discussed methods for A549 cells (Perez et al. 2011). Hyperoxia exposure was stopped at different times (18 h for apoptosis analysis, 2 days for p‐Akt analysis, and 4 days for EMT marker analysis and RNA isolation).

### Immunocytochemistry

RLE cells were grown in glass coverslips and exposed to room air or hyperoxia for 18 h (for cleaved caspase‐3 or TUNEL staining) or 4 days (E‐cadherin staining). For E‐cadherin staining, coverslips were fixed in methanol at −20°C for 2 min, followed by 3 washes in PBS and blocking for 20 min in 5% BSA in PBS. Mouse anti‐E‐cadherin antibody (1:400) was used in 1% BSA in PBS for 1 h at room temperature, followed by 3 washes in PBS, secondary goat antimouse‐Alexafluor 594 (Molecular Probes) for 1 h at room temperature in the dark, three more washes in PBS and then coverslips were mounted onto slides using Prolong Gold antifade with DAPI. For cleaved caspase‐3 staining, a protocol provided by Cell Signaling was carefully followed, which included modifications in blocking solution and antibody dilution, and an overnight staining step with the rabbit monoclonal antibody against cleaved caspase‐3. Stained cells were observed under an Olympus BX60 fluorescence microscope, and pictures were taken.

### TUNEL Staining

For RLE coverslips and alveolar epithelial type II cell cytospins, the In situ Cell Death detection kit with fluorescein from Roche was used. For lung section staining, the Promega DeadEnd fluorometric detection kit was used (Madison, WI, US). In both cases, manufacturer's instructions were carefully followed for optimal results.

### Statistical analysis

Results are expressed as mean ±  SD of data obtained. Statistical analysis was performed with Student's t‐test for paired comparisons and analysis of variance (ANOVA) was used to analyze differences between experimental groups. A value of *P *<* *0.05 was considered significant.

## Results

### RLE overexpressing TRIP‐1 are resistant to hyperoxia‐induced epithelial injury

**Figure 1:**
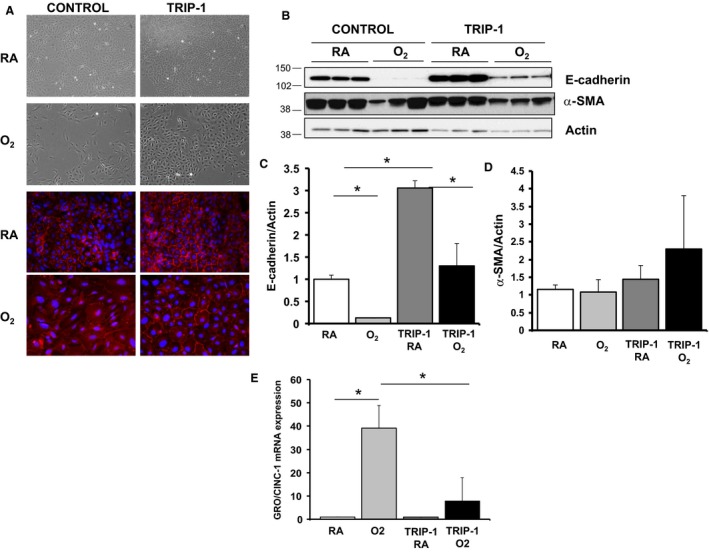
RLE cells overexpressing TRIP‐1 resist hyperoxia‐induced reduction in E‐cadherin expression and IL‐8 expression. (A) CONTROL cells exposed to 85% O_2_ for 4 day, showing fewer cell‐to‐cell connections, consistent with a mesenchymal cell type, and decreased E‐cadherin staining (red). RLE cells overexpressing V5‐TRIP‐1 (TRIP‐1) maintain cell‐to‐cell connections and show more E‐cadherin staining. (B) Expression of E‐cadherin and α‐SMA assessed by Western blot. Cells were exposed to normoxia (RA) or 85% O_2_ for 4 day, after which lysates were made; (C) Quantification of E‐cadherin from (B); *P < 0.05 (*n* = 3). (D) Quantification of α‐SMA from B); *P < 0.05 (*n* = 3). (E) GRO/CINC‐1 mRNA expression in control or TRIP‐1 overexpressing RLEs, exposed to room air (RA) or 95% O_2_ for 3 days; *P < 0.05 (*n* = 3).RLE, Rat lung epithelial

Hyperoxia exposure leads to epithelial injury, loss of epithelial integrity, and possibly epithelial transdifferentiation. To determine whether TRIP‐1 overexpression in RLE cells protects against hyperoxia‐induced epithelial injury, we exposed them to hyperoxia. We observed a loss of cell‐to‐cell interaction in control RLE, whereas TRIP‐1 overexpressing RLE maintained robust cell‐to‐cell interaction consistent with an epithelial phenotype (Fig. 1A). With respect to E‐cadherin, we observed higher basal E‐cadherin expression in the TRIP‐1 overexpressing RLE relative to controls. Oxygen exposure caused a reduction in E‐cadherin expression. However, the TRIP‐1 overexpressing RLE were able to maintain their much higher E‐cadherin expression (Fig. 1B and C). Interestingly, we observed insignificant α‐SMA expression in hyperoxia, suggesting no mesenchymal transition (Fig. 1B and D).

Epithelial cell injury can lead to secretion of specific inflammatory cytokines. IL‐8, a proinflammatory chemokine thought to enhance inflammatory migration and phagocytosis is one of these particular cytokines. Interestingly, hyperoxia increased GRO/CINC‐1 (rat homolog to human IL‐8) expression in control RLE but the RLE cells overexpressing TRIP‐1 showed only a mild increase in GRO/CINC‐1 expression (Fig. 1E).

**Figure 2:**
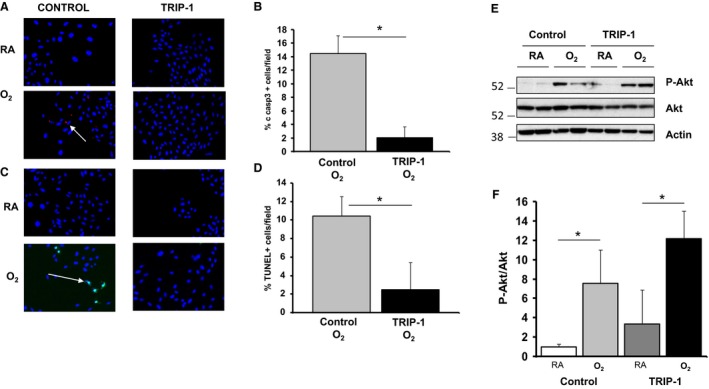
RLE cells overexpressing TRIP‐1 are protected from hyperoxia‐induced apoptosis. (A) Rat lung epithelial cells were exposed to RA or 85% O_2_ for 18 h. Treatment with 85% O_2_ for 18 h showed positive cleaved caspase 3 staining (white arrow) in control cells, but not in TRIP‐1 overexpressing RLE cells (TRIP‐1). (B) Quantification of % cells stained positive for cleaved caspase 3 in hyperoxia‐exposed cells; *P < 0.05 (*n* = 3). (C) The same cells also show TUNEL‐positive signal (white arrow), which is absent if the RLE cells overexpress TRIP‐1 (TRIP‐1). (D) Quantification of % cells stained positive for TUNEL in hyperoxia‐treated cells; *P < 0.05 (*n* = 3). (E) Cells were exposed to RA or 85% O_2_ for 2 day, after which lysates were made and analyzed by Western blot. RLE cells overexpressing TRIP‐1 (TRIP‐1) have increased hyperoxia‐induced phosphorylation of prosurvival protein Akt. (F) Quantitation of *P*‐Akt/Akt from (E); *P* < 0.05 (*n* = 3). RLE, Rat lung epithelial

Lung epithelial cells are known to have a robust antioxidant system, however, prolonged exposure to hyperoxia can result in apoptosis(Crapo et al. 1980; Barazzone et al. 1998). To determine whether TRIP‐1 overexpression protects RLE against hyperoxia‐induced apoptosis, we exposed the RLE overexpressing TRIP‐ 1 and controls to hyperoxia. In the control RLE, we observed higher levels of cleaved caspase‐3 following oxygen exposure than in TRIP‐1 overexpressing RLEs (14.5 ± 2.6% vs. 2.1 ± 1.6% *P *<* *0.05) and more TUNEL staining (10.5 ± 2.1% vs. 2.5 ± 2.9% *P *<* *0.05) (Fig. 2A–D). To determine whether TRIP‐1‐mediated reduction in apoptosis could be attributed to Akt activation, we assessed phosphorylated Akt (p‐Akt) levels. Hyperoxia led to p‐Akt induction in both controls and TRIP‐1 overexpressing RLE. However, the RLE overexpressing TRIP‐1 showed higher p‐Akt expression at baseline and following oxygen exposure (Fig. 2E and F). These findings suggest that during acute hyperoxia exposure, TRIP‐1 overexpression in lung epithelial cells preserves lung epithelial cell phenotype, reduces GRO/CINC‐1 expression, and resists apoptosis in association with increased p‐AKT expression during acute hyperoxia exposure without overt changes in the expression of mesenchymal markers.

### Lung TRIP‐1 expression decreases postnatally in WT mice; TRIP‐1^AECTg+^ mice have normal lung development

**Figure 3:**
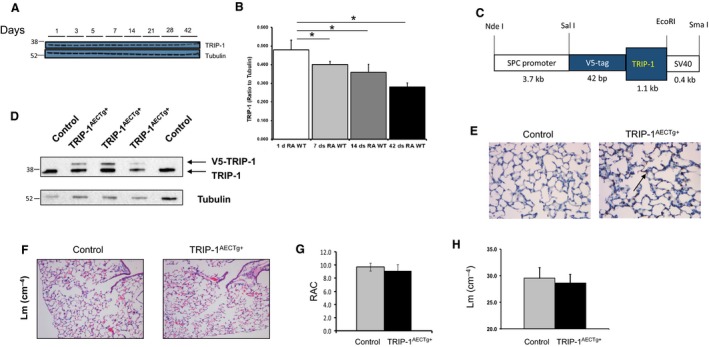
TRIP‐1 expression decreases in an age‐dependent manner in C57Bl/6 and SPC‐ TRIP‐1^AECTg+^ expressing mice with targeted, specific overexpression in type II epithelial cells have normal lung development. (A) TRIP‐1 expression in control mouse lungs exposed to room air assessed by Western blot at specific days of life. (B) Quantification of TRIP‐1 normalized to Tubulin from (A); *P < 0.05 (*n* = 3). (C) Construct used for targeted expression in TRIP‐1^AECTg+^ mouse model. (D) Western‐blot analysis of lung tissue shows expression of TRIP‐1, appearing as a doublet reflecting endogenous and overexpressed TRIP‐1 protein. (E) V5 immunohistochemistry of 4‐week‐old mice, detecting expression of the V5‐TRIP‐1 construct (brown) in lung epithelial cells (black arrow). All experiments were performed 3 times. *P* < 0.05. (F) Representative H&E stained sections (×100) from 4‐week‐old control and TRIP‐1^AECTg+^ mice. (G) Radial Alveolar Count of controls and TRIP‐1^AECTg+^ mice. (H) Mean Linear Intercepts (Lm) of controls and TRIP‐1^AECTg+^ mice.

We have previously reported that higher TRIP‐1 expression in fetal versus adult fibroblasts contributes to different contractility, and that epithelial cells overexpressing TRIP‐1 resist TGFβ‐mediated cellular responses(Navarro et al. 2009; Perez et al. 2011). To determine whether TRIP‐1 expression is developmentally expressed in the lung, we collected lung samples from WT mice from postnatal day 1 through 48. Interestingly, TRIP‐1 expression was inversely correlated with increasing age, with adult mice expressing TRIP‐1 at 50% of levels expressed in mouse pups. (Fig. 3A and B). This finding, taken together with our previous publications, suggests TRIP‐1 is a developmentally expressed protein and may contribute to age‐specific responses to acute hyperoxic epithelial injury and subsequent repair. To further assess TRIP‐1′s specific role in epithelial lung injury, and to validate our in vitro data showing a protective role of TRIP‐1 in hyperoxic injury, we developed an in vivo model using the SPC‐promoter to overexpress TRIP‐1 in type II epithelial cells (Fig. 3C). TRIP‐1^AECTg+^ mice and age‐matched controls were killed at 4‐5 weeks of age to assess for specificity of TRIP‐1 overexpression and to evaluate the lung phenotype. The lungs of the TRIP‐1^AECTg+^ mice exhibited V5‐TRIP‐1 (Fig. 3D and E) and showed normal alveolarization patterns (Fig. 3F–H). These mice did not develop any respiratory difficulty throughout their life cycle and confirmed specific TRIP‐1 overexpression in type II epithelial cells.

### TRIP‐1^AECTg+^ mice have reduced hyperoxia‐induced macrophage accumulation, IL‐8 expression, TGFβ activation, and a survival benefit in prolonged hyperoxia exposure

**Figure 4:**
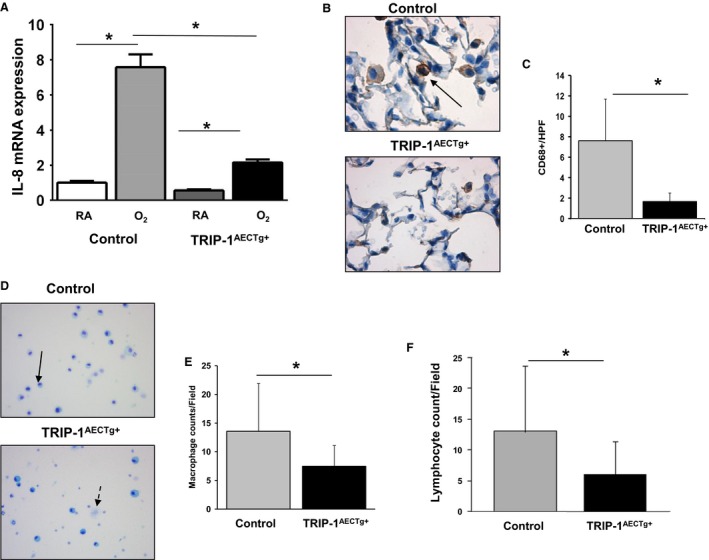
TRIP‐1^AECTg+^ mice have lower IL‐8 expression, less macrophage infiltration, and enhanced E‐cadherin expression following hyperoxia exposure. (A) KC mRNA expression in lung tissue from TRIP‐1^AECTg+^ mice and controls, RA or 95% O_2_ for 4 day, *P < 0.05 (*n* = 4 mice/group). (B) CD68 (black arrow) of 5 *μ*m lung sections from 4‐week‐old age mice exposed to >95% O_2_ for 4 days. Lung sections stained for Images from hyperoxia‐exposed mice. (C) Quantification of the average number of CD68 positive cells/HPF at 600X; *P < 0.05 (*n* = 5 mice/group). (D) bronchoalveolar lavage fluid stained with DiffQuik (solid black arrow pointing at macrophage and dashed black arrow pointing at lymphocyte). (E) Quantification of macrophages by average number of macrophages/field; *P < 0.05 (*n* = 5 mice/group). (F) Quantification of lymphocytes by average number of lymphocytes/field; *P < 0.05 (*n* = 11 mice/group).

**Figure 5:**
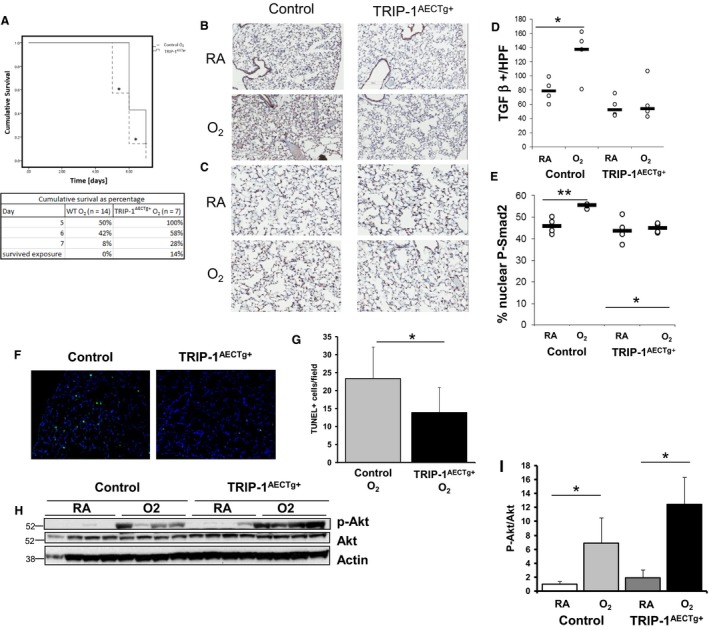
TRIP‐1^AECTg+^ mice have less hyperoxia‐induced TGFβ1 staining, nuclear pSMAD2 staining, TUNEL staining but higher p‐Akt following hyperoxia exposure. Control and TRIP‐1^AECTg+^ mice exposed to >95% O_2_ for upto 7 days at 4 weeks of age. (A) Kaplan–Meier Survival Curve *P < 0.05 (*n* = 14 controls and *n* = 7 TRIP‐1^AECTg+^ mice). Remaining figures based off control and TRIP‐1^AECTg+^ mice exposed to either RA or >95% O_2_ for 4 days at 4 weeks of age. (B) TGFβ1 immunohistochemical stain. (C) Average number of TGFβ1 staining positive cells/field at 200X; *P < 0.05. (D) pSMAD2 immunohistochemical stain. (E) % of nuclear pSMAD2/field at 200X; **P < 0.01 (*n* = 4 mice/group). (F) TUNEL staining from TRIP‐1^AECTg+^ and controls. (G) TUNEL staining/field; *P < 0.05 (*n* = 4 mice/group). (H) Western blot from TRIP‐1^AECTg+^ mice‐ and age‐matched controls for p‐Akt and Akt; (I) graph depicting difference in p‐Akt normalized to Akt; *P < 0.05 (*n* = 4 mice/group).

Adult mice exposed to hyperoxia show significant epithelial cell injury, apoptosis, inflammatory cell (specifically, macrophage) migration into alveolar spaces, and mesenchymal cell proliferation(Nagato et al. 2012). We exposed 4‐week‐old TRIP‐1^AECTg+^ mice and age‐matched controls to 4 days of > 95% O_2_. We observed hyperoxia increased KC (mouse homolog to human IL‐8) induction in control mouse whole lung that was negated in the TRIP‐1^AECTg+^ mice (Fig. 4A). This elevated KC expression correlated with accumulation of alveolar macrophages. While at baseline we did not observe a difference in the number of alveolar macrophages (2.69 ± 0.32 vs. 3.21 ± 0.3 per HPF p = NS), we did observe more alveolar macrophages in control lungs exposed to hyperoxia when compared with TRIP‐1^AECTg+^ (7.6 ± 4.1 vs. 1.6 ± 0.8 per HPF *P *<* *0.05) (Fig. 4B and C). In addition, we observed higher macrophage and lymphocyte counts in the bronchoalveolar lavage fluid (BALF) of the controls exposed to hyperoxia (13.6 ± 8.4 vs. 7.5 ± 3.7 per field; *P *<* *0.05 and 13.1 ± 10.5 vs. 6.1 ± 5.3; *P *<* *0.05, respectively) (Fig. 4D–F). Additionally, we observed TRIP‐1^AECTg+^ mice show a survival benefit in prolonged hyperoxia exposure. (Fig. 5A). These findings suggest epithelial‐specific TRIP‐1 overexpression attenuates KC expression, prevents macrophage invasion into the lung, and provides a survival benefit during hyperoxia exposure.

TGFβ‐induced epithelial damage/apoptosis has been implicated as a factor contributing to injury in HALI(Vyas‐Read et al. 2014). To determine the role epithelial‐specific TRIP‐1 plays in hyperoxia‐induced TGFβ activation, we looked at TGFβ activation in TRIP‐1^AECTg+^ mice and similarly aged control mice. Hyperoxia increased TGFβ1 and nuclear p‐SMAD2 staining in control mice, whereas TRIP‐1^AECTg+^ mice did not show an increase in TGFβ1 and p‐SMAD2 staining (Fig. 5A–D). This suggests that TRIP‐1 overexpression in lung epithelial cells protects against hyperoxia‐induced TGFβ activation observed in HALI.

### Hyperoxia‐induced activation of Akt is potentiated, whereas E‐cadherin expression is preserved in hyperoxia‐exposed lung epithelial cells overexpressing TRIP‐1

To further determine epithelial‐specific TRIP‐1 overexpression in epithelial injury, we evaluated the TRIP‐1^AECTg+^ mice for apoptosis. Similar to our observations using RLE cell in vitro, we observed that hyperoxia increased TUNEL staining in controls, whereas the TRIP‐1^AECTg+^ mice showed less TUNEL staining (23.4 ± 8.8 vs. 13.2 ± 6.9 cells per field *P *<* *0.05) (Fig. 5F and G). While hyperoxia exposure increased p‐Akt activity in controls and TRIP‐1^AECTg+^ mice, the activation of Akt in the TRIP‐1^AECTg+^ mice was significantly higher (Fig. 5H and I).

**Figure 6:**
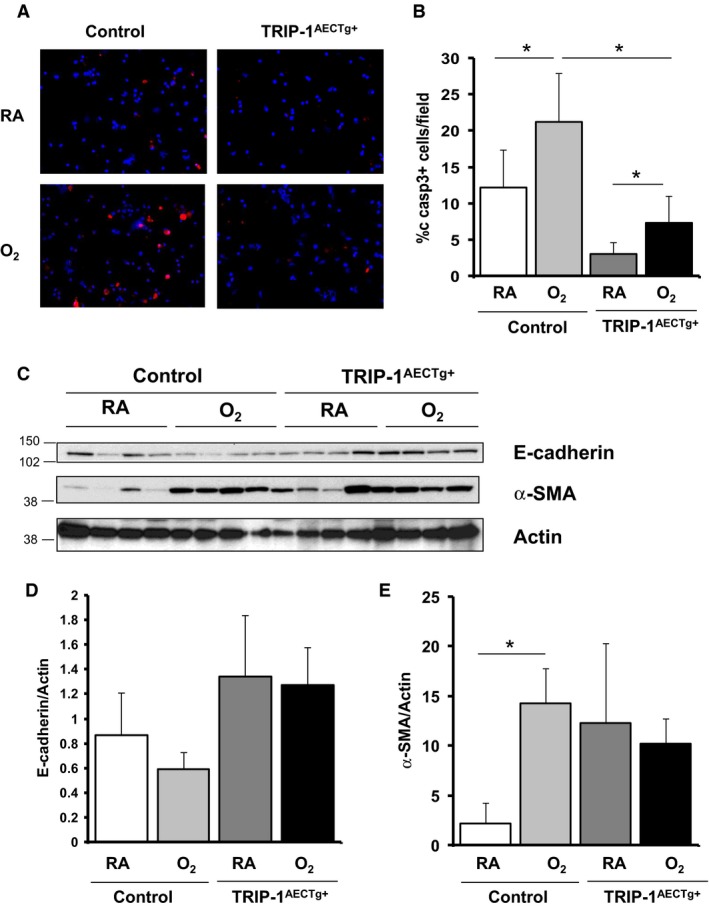
Alveolar epithelial cells from TRIP‐1^AECTg+^ mice have less hyperoxia‐induced cleaved caspase‐3 and higher E‐cadherin than those from control mice. Type II AEC were isolated from 4‐week‐old mice exposed to RA or >95% O_2_ for 3 day. (A) Cleaved caspase‐3 staining on isolated type II AEC for apoptosis analysis; (B) % cleaved caspase‐3 positive cells in the type II cell isolated population; *P < 0.05 (*n* = 4). (C) Western blot of E‐cadherin and α‐SMA in isolated type II AEC; (D) quantification of E‐cadherin in (C); p = NS (*n* = 4), (E) quantification of α‐SMA in (C); *P < 0.05 (*n* = 4).

To further delineate that these findings were specific to type II epithelial cells, we isolated type II lung epithelial cells from TRIP‐1^AECTg+^ mice and control mice exposed to RA or hyperoxia. In both TRIP‐1^AECTg+^ and controls cells, cleaved caspase‐3 staining increased when animals were exposed to hyperoxia. However, in cells from hyperoxia‐exposed mice, cleaved caspase‐3 staining was significantly less in TRIP‐1^AECTg+^ cells compared to control cells (Fig. 6A and B). E‐cadherin expression was greater in RA and oxygen‐exposed type II cells isolated from TRIP‐1^AECTg+^ mice compared to RA cells from control mice. Although we saw decreased E‐cadherin expression in control cells exposed to hyperoxia, the difference was not statistically significant. Interestingly, there was a very significant induction of α‐SMA expression in control cells when animals were exposed to oxygen, not seen in cells from transgenic animals, although those cells had a higher basal expression of the protein (Fig. 6C–E). The data suggests that TRIP‐1 attenuates type II cell apoptosis in association with preservation of epithelial marker E‐cadherin without significantly altering α‐SMA expression in hyperoxia.

## Discussion

In this work, we report on a novel role for TRIP‐1 in developmentally expressed responses to hyperoxia‐mediated epithelial injury. First, we show TRIP‐1 is highly expressed in mouse pup lungs and that levels decline with advancing age such that adult lungs have only about 50% the level of pup lungs. Second, we generated a transgenic TRIP‐1 mouse with specific overexpression in lung epithelial cells. This mouse showed normal lung development and was found to be relatively resistant to HALI. Of particular note, these transgenic mice showed very high levels of phospho‐Akt in the lung and high E‐cadherin expression in the type II AEC. Finally, in parallel experiments using RLE cells engineered to overexpress TRIP‐1, we confirmed our in vivo findings that TRIP‐1 overexpression instills resistance to apoptosis signaling, leads to high phospho‐Akt levels, and maintains higher E‐cadherin expression in hyperoxia. These findings are important because they suggest that TRIP‐1 expression in type II AEC may be a factor in age‐specific responses during initiation of hyperoxia‐induced epithelial injury and that high TRIP‐1 levels attenuate hyperoxia‐induced apoptosis and epithelial injury observed in HALI. While the role type II AEC injury plays in HALI is well established, this study exposes TRIP‐1 as a developmentally expressed factor mitigating epithelial apoptosis and thereby maintaining epithelial integrity.

Type II AEC are essential for maintaining homeostatic pulmonary function under normal physiological conditions and act as progenitor cells for the type I AEC. Hyperoxia induces excessive TGFβ‐mediated epithelial cell death, either by apoptosis or necrosis. Interestingly, our data demonstrate that type II epithelial cells, both RLE and isolated primary mouse type II AEC overexpressing TRIP‐1, resist hyperoxia‐induced apoptosis, as observed with both TUNEL and cleaved capase‐3 staining.

Protein kinase B/Akt is an antiapoptotic protein thought to mediate apoptotic signaling via multiple pathways. (Lu et al. 2001) have reported that virally transfected activated Akt protects epithelial cells from hyperoxia lung injury in the mouse lung. Akt activation occurs through many pathways including the non‐SMAD TGFβ signaling pathway (Zang 2009). We observed higher basal p‐Akt activity in TRIP‐1 overexpressing RLE cells and isolated primary mouse type II AEC in our model, suggesting that TRIP‐1 may play a central role in activating Akt. Higher p‐Akt levels were protective in our model. These observations, taken together with higher E‐cadherin expression, suggest TRIP‐1 maintains epithelial integrity.

High E‐cadherin expression is a hallmark feature of epithelial cells. E‐cadherin is a transmembrane glycoprotein that mediates specific cell‐cell adhesion in a Ca^++^‐dependent manner. It interacts with numerous signaling pathways, promoting cell‐cell interaction, while inhibiting apoptosis signaling and cell differentiation. Pece et al. (1999) previously reported that E‐cadherin, through cell‐cell interactions, regulates phospho‐Akt via enhanced phosphatidylinositol 3 kinase (PI3K) activity, and Andl et al. (2006) demonstrated that E‐cadherin can bind directly to the ectodomain of type II TGFβ receptor, inferring a regulatory role on TGFβ mediated responses. These reports are of particular interest due to our observations of higher E‐cadherin expression and phospho‐Akt in cells overexpressing TRIP‐1 suggesting a potential E‐cadherin/Type II TGFβ receptor/Akt/TRIP‐1 interaction which will require further investigation.

It is well established that the loss of E‐cadherin is associated with increased motility, loss of cell adhesion, and a promesenchymal phenotype in type II AEC. Epithelial‐mesenchymal transition plays an important role during early embryonic growth and resolution of lung injury (Crosby and Waters 2010). Excessive TGFβ signaling promotes EMT in the mature lung, contributing to the development of fibrosis, but the role that EMT plays in HALI remains controversial (Liu and Desai 2015). In our hyperoxia exposure model, we did not detect full EMT changes. Control RLE and isolated type II AEC showed reduced E‐cadherin expression, and while isolated type II AEC showed elevated α‐SMA, we could not detect a similar increase in RLE following hyperoxia exposure. In the TRIP‐1 overexpressing RLE and isolated type II AEC, we observed higher basal E‐cadherin and α‐SMA expression. Additionally, these TRIP‐1 overexpressing cells showed reduced E‐cadherin expression following hyperoxia but E‐cadherin levels were much higher than hyperoxia‐exposed controls implies level of TRIP‐1 expression may influence some EMT signaling. Overall these observations provide evidence that in hyperoxia, TRIP‐1 maintains epithelial integrity by fostering higher E‐cadherin expression.

A hallmark feature of hyperoxia‐induced lung injury in mice is the accumulation of inflammatory cells, specifically macrophages, within alveolar spaces following epithelial cell injury and apoptosis that leads to death. We demonstrated that epithelial‐specific overexpressing TRIP‐1^AECTg+^ mice had fewer macrophages in alveolar spaces and in BALF following hyperoxia exposure and these mice also showed a survival benefit in prolonged hyperoxia exposure. These observations are another piece of evidence suggesting TRIP‐1 overexpression protects against HALI. Epithelial injury and loss of integrity play central roles in the accumulation and recruitment of inflammatory cells and specific cytokine release, such as KC (mouse homolog to human IL‐8). In our model, we did not demonstrate universal differences in inflammatory cytokines; however, we did detect lower KC expression in the hyperoxia‐exposed TRIP‐1 overexpressing mice. IL‐8 and its homologs are proinflammatory chemokine produced by activated macrophages, and other cell types such as injured epithelial cells, that enhance inflammatory cell migration and phagocytosis. Interestingly, we observed TRIP‐1‐dependent suppression of hyperoxia‐induced GRO/CINC‐1 expression in RLE, suggesting that in our model, the GRO/CINC‐1 expression occurred through epithelial injury. Reports by other researchers have associated higher IL‐8 expression with EMT in hepatocytes and, in combination with TGFβ activation, impaired fluid transport in rat epithelial cells, contributing to the development of acute respiratory distress syndrome, a disease exacerbated by hyperoxia (Wagener et al. 2015; Long et al. 2016). Taken together with these other reports, our findings infer that TRIP‐1 mediates HALI by maintaining epithelial integrity by preventing KC expression and the accumulation/activation of macrophages within the alveolar spaces.

In summary, we found that TRIP‐1 expression decreases with age and TRIP‐1 overexpression in type II AEC maintains epithelial integrity by sustaining E‐cadherin expression and preventing apoptosis via phospho‐Akt signaling, suggesting a protective role for TRIP‐1 in type II AEC. Mice overexpressing TRIP‐1 in their lung type II alveolar epithelial cells showed normal lung development, increased phospho‐AKT and E‐cadherin, and were resistant to HALI, as evidenced by less TGFβ activation, apoptosis, alveolar macrophage influx, and KC expression, suggesting a TRIP‐1‐mediated molecular pathway affording protection against acute epithelial lung injury. Future studies will focus on mechanisms by which TRIP‐1 expression protects epithelial cells against hyperoxia injury, while also exploring TRIP‐1′s role in hyperoxia‐mediated abnormal postnatal lung development in mouse models of bronchopulmonary dysplasia.

## Conflict of Interest

Authors do not have any conflicts of interest.
